# Patterns of Long COVID Symptoms: A Multi-Center Cross Sectional Study

**DOI:** 10.3390/jcm11040898

**Published:** 2022-02-09

**Authors:** Dana Yelin, Ili Margalit, Mayssam Nehme, Jaume Bordas-Martínez, Francesco Pistelli, Dafna Yahav, Idris Guessous, Xavier Durà-Miralles, Laura Carrozzi, Irit Shapira-Lichter, Pauline Vetter, Dolores Peleato-Catalan, Giusy Tiseo, Eytan Wirtheim, Laurent Kaiser, Carlota Gudiol, Marco Falcone, Leonard Leibovici, on behalf of the LongCOV Research Group

**Affiliations:** 1 COVID Recovery Clinic, Rabin Medical Center, Beilinson Hospital, Petah-Tikva 4941492, Israel; ilimargalit@gmail.com; 2 Faculty of Medicine, Tel Aviv University, Ramat Aviv, Tel Aviv 6910203, Israel; dafna.yahav@gmail.com (D.Y.); iritlichter@yahoo.com (I.S.-L.); 3 Infectious Diseases Unit, Rabin Medical Center, Beilinson Hospital, Petah-Tikva 4941492, Israel; 4 Division and Department of Primary Care Medicine, Geneva University Hospitals, 1211 Geneva, Switzerland; Mayssam.Nehme@hcuge.ch (M.N.); Idris.Guessous@hcuge.ch (I.G.); 5 Pulmonology Department, Bellvitge University Hospital, 08907 Barcelona, Spain; jbordas@bellvitgehospital.cat; 6 Infectious Diseases Department, Bellvitge University Hospital, Institute for Biomedical Research (IDIBELL), University of Barcelona, Hospitalet de Llobregat, 08907 Barcelona, Spain; laura.carrozzi@unipi.it (L.C.); carlotagudiol@gmail.com (C.G.); 7 Department of Surgical, Medical, and Molecular Pathology and Critical Care Medicine, University of Pisa, 56126 Pisa, Italy; f.pistelli@ao-pisa.toscana.it; 8 Pulmonary Unit, Cardiothoracic and Vascular Department, University Hospital, 56124 Pisa, Italy; 9 Faculty of Medicine, University of Geneva, 1205 Geneva, Switzerland; xavierduramiralles@gmail.com; 10 Spanish Network for Research in Infectious Diseases (REIPI), Instituto de Salud Carlos III, 08003 Madrid, Spain; 11 Functional MRI Center, Rabin Medical Center, Beilinson Hospital, Petah-Tikva 4941492, Israel; 12 Division of Infectious Diseases, Geneva University Hospitals, 1211 Geneva, Switzerland; Pauline.Vetter@hcuge.ch (P.V.); Laurent.Kaiser@hcuge.ch (L.K.); 13 Ramona via Primary Care Center, El Prat de Llobregat, 08820 Barcelona, Spain; mpeleato@ambitcp.catsalut.net; 14 Infectious Diseases Unit, Department of Clinical and Experimental Medicine, University of Pisa, 56126 Pisa, Italy; tiseogiusy@gmail.com (G.T.); marco.falcone@unipi.it (M.F.); 15 Management, Rabin Medical Center, Petah-Tikva 4941492, Israel; eytanwir@clalit.org.il; 16 Infectious Diseases Unit, Hospital Duran y Reynals, Institut Català d’Oncologia (ICO), IDIBELL, 08908 Barcelona, Spain; 17 Centro de Investigación Biomédica en Red de Enfermedades Infecciosas (CIBERINFEC), Instituto de Salud Carlos III, 08003 Madrid, Spain; 18 Research Authority, Rabin Medical Center, Beilinson Hospital, Petah-Tikva 4941492, Israel; leibovic@tauex.tau.ac.il

**Keywords:** COVID-19, post-COVID, long lasting symptoms, SARS-CoV-2

## Abstract

Background: Long COVID has become a burden on healthcare systems worldwide. Research into the etiology and risk factors has been impeded by observing all diverse manifestations as part of a single entity. We aimed to determine patterns of symptoms in convalescing COVID-19 patients. Methods: Symptomatic patients were recruited from four countries. Data were collected regarding demographics, comorbidities, acute disease and persistent symptoms. Factor analysis was performed to elucidate symptom patterns. Associations of the patterns with patients’ characteristics, features of acute disease and effect on daily life were sought. Results: We included 1027 symptomatic post-COVID individuals in the analysis. The majority of participants were graded as having a non-severe acute COVID-19 (N = 763, 74.3%). We identified six patterns of symptoms: cognitive, pain-syndrome, pulmonary, cardiac, anosmia-dysgeusia and headache. The cognitive pattern was the major symptoms pattern, explaining 26.2% of the variance; the other patterns each explained 6.5–9.5% of the variance. The cognitive pattern was higher in patients who were outpatients during the acute disease. The pain-syndrome pattern was associated with acute disease severity, higher in women and increased with age. The pulmonary pattern was associated with prior lung disease and severe acute disease. Only two of the patterns (cognitive and cardiac) were associated with failure to return to pre-COVID occupational and physical activity status. Conclusion: Long COVID diverse symptoms can be grouped into six unique patterns. Using these patterns in future research may improve our understanding of pathophysiology and risk factors of persistent COVID, provide homogenous terminology for clinical research, and direct therapeutic interventions.

## 1. Background

Persistent symptoms following coronavirus disease 2019 (COVID-19) have been described in a significant percentage of post-COVID individuals. The only established correlates identified thus far are the severity of the acute disease and a higher preponderance in women [1,2,3,4]. Other potential risk factors such as age have been described in some studies, but not in others [1,3,5].

Similar to the acute disease, which manifests in multiple ways, post-COVID individuals report a multitude of symptoms, involving different organ systems [6]. Whereas, in the acute disease, several pathophysiological mechanisms have already been identified (i.e., direct viral induced damage, microthrombi and immune mediated damage), the underlying mechanisms behind long COVID are still unknown and are likely to be multifactorial. Aggregating all individuals with long COVID under a single entity might be misleading for our understanding of the pathophysiology, risk factors and potential interventions for these individuals. Assigning these symptoms to unique patterns might advance future research and a personalized treatment approach [7].

In this study, we aimed to determine patterns of symptoms among convalescing COVID-19 individuals.

## 2. Methods

### 2.1. Study Design, Participants and Data Collection

The study was a multi-center cohort study, conducted in the multi-disciplinary long-COVID clinics in four countries (Israel, Switzerland, Spain and Italy). Data were collected either at the first clinic visit (Israel, Italy, Spain) or by telephone interview (Switzerland, Spain). Adult (age ≥ 18 years) individuals visiting the clinics between May 2020 and March 2021 reporting on at least one persisting symptom, at least 30 days after a PCR-proven diagnosis of COVID-19 were consecutively included (for additional information regarding the clinics and respective structured follow-up programs—see Supplementary Material). Each patient was interviewed, using a structured questionnaire (grading 14 symptoms subjectively according to severity on a 0–3 scale, representing no, mild, moderate and severe impact on daily life respectively). Graded symptoms were: fatigue, dyspnea, cough, chest pain, palpitations, insomnia, memory impairment, concentration difficulties, myalgia, arthralgia, paresthesia (numbness or tingling), headache, anosmia/dysgeusia, and emotional distress. Data regarding part of the patients from the Swiss cohort were previously reported [8]. Pulmonary function testing was completed in three of the four centers (Israel, Spain, Italy).

The study was approved by the local ethics committee of each participating center (approval numbers: Israel 0458-20-RMC; Italy CEAVNO n. 1768; Spain PR374/20; Switzerland CER 2020-01273). All patients signed or gave oral informed consent prior to participation.

### 2.2. Statistical Analysis

A principal component analysis of the 14 reported symptoms was conducted in order to identify key patterns of symptoms. We used a varimax rotation. The symptoms were tested for factorability using correlates above 0.3 and Kaiser–Meyer–Olkin measure of sampling adequacy of over 0.6. Eigenvalues above 0.9 were used to select the number of factors. Loading of 0.4 and up was considered as significant.

We looked for associations of the patterns with the patients’ age, gender, underlying comorbidities, and features of the acute disease (hospitalization, severe disease according to WHO classification [9], and acute symptoms). We also examined whether the factors were related to return to work and return to the pre-infection level of physical activity. Student’s *t*-tests and analysis of variance (ANOVA) were used for comparing the values of the factors across categories; Spearman’s rank correlation coefficient was implemented for testing the correlation of factors with continuous variables. We used the Bonferroni correction to adjust *p* values for multiple hypotheses and reported the adjusted *p* values. Data analysis was undertaken using IBM SPSS version 27 (Armonk, New York, NY, USA).

## 3. Results

From the four centers, 1737 participants were recruited. Out of these, 1033 (59.5%) reported ongoing symptoms, however, 6 had incomplete responses regarding one or more of the 14 symptoms and were excluded. In total, 1027 persistently symptomatic adult patients were included in the study (see Supplemental Figure S1). The median interval from onset of acute COVID-19 to the interview was 123 days (interquartile range 80–204 days). Included patients had acute COVID-19 from March 2020 to March 2021. Five hundred fifty nine participants (54.4%) were women and 337 (32.8%) were hospitalized during the acute disease. Patient characteristics and the prevalence of each persistent symptom in this cohort are presented in Table 1. Three hundred twenty six individuals (31.7%) reported worse physical activity status compared to pre-infection state, and 131 (of the 735 for whom data were available, 17.8%) were unable to return to their former work because of ongoing symptoms at the time of the clinic visit.

**Figure 1:**
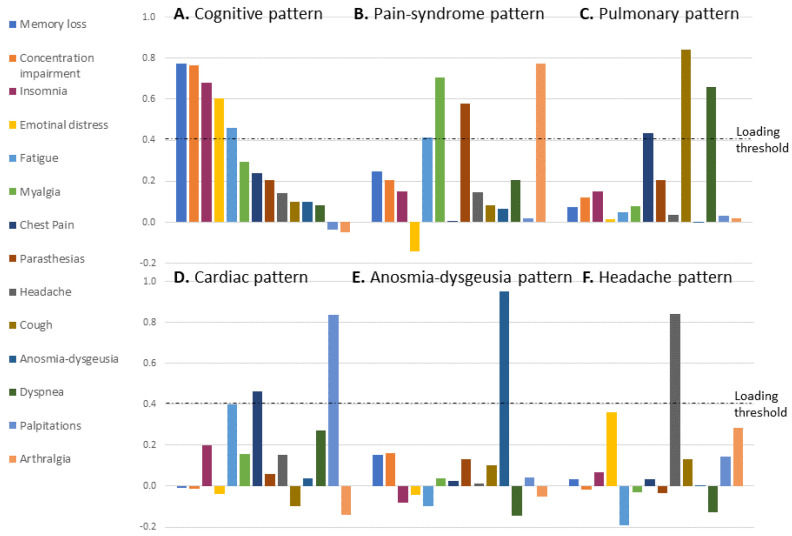
Loading of the different symptoms in each symptom pattern. (**A**) Cognitive-emotional pattern combining impaired cognitive functions, emotional distress, insomnia and fatigue (explaining 26.2% of variance); (**B**) Pain syndrome pattern combining myalgia, arthralgia, paresthesias and fatigue (9.5% of variance) (**C**) Pulmonary pattern combining dyspnea, cough and chest pain (8.5% of variance); (**D**) Cardiac—combining palpitations or tachycardia with chest pain (7.1% of variance), fatigue with a loading of 0.39 is under the threshold; (**E**) Anosmia-dysgeusia pattern—isolated anosmia and dysgeusia (6.8% of variance); (**F**) Headache pattern—isolated headache (6.5% of variance); The 6 factors in total explain 64.6% of variance between the recoverees; KMO: 0.811, Bartlett’s test of sphericity chi-square: 2682.51, *p* < 0.001.

Using factor analysis, we identified 6 different patterns of the 14 symptoms (Figure 1, for exact loading data see Supplemental Table S1). The first was a cognitive-emotional pattern combining complaints of impaired concentration, memory loss, emotional distress and insomnia. The second was indicative of pain manifestations: myalgia, arthralgia and, to a lesser extent, paresthesias and fatigue. The third was a pulmonary pattern, combining dyspnea, cough and chest pain. The fourth pattern (cardiac) consisted of a combination of palpitations or tachycardia with chest pain. The fifth and sixth patterns were anosmia and/or dysgeusia and isolated headache, respectively. The cognitive-emotional pattern was the major symptoms pattern, explaining 26.2% of the variance; each of the other patterns explained 6.5–9.5% of the variance. Cumulatively, all patterns explained 64.6% of the variance. On four sensitivity analyses, excluding one of the centers successively, similar patterns were achieved each time; the differences manifested in the order of the factors only.

Associations of the various patterns with demographics, comorbidities and manifestations of the acute disease are detailed in Table 2. The cognitive-emotional pattern was associated with features of the acute disease: outpatient treatment (mean 0.1 (standard deviation 1.03) vs. −0.15 (1.00), *p* < 0.001), fatigue (0.25 (1.13) vs. −0.37 (0.75), *p* < 0.001), myalgia (0.29 (1.18) vs. −0.22 (0.84), *p* < 0.001) and anosmia (0.34 (1.17 vs. −0.18 (0.90), *p* < 0.001). The pain-syndrome pattern was associated with the severity of the acute disease (0.22 (0.98) vs. −0.08 (1.00), *p* < 0.001) and was higher in women (0.11 (1.08) vs. −0.13 (0.88), *p* < 0.001). The pulmonary pattern was associated with prior lung disease (0.41 (1.24) vs. −0.03 (0.97), *p* < 0.001), severe acute disease (0.20 (0.98) vs. −0.07 (0.97), *p* < 0.001), inpatient treatment (0.17 (0.98) vs. −0.07 (1.01), *p* < 0.001), and dyspnea (0.33 (1.16 vs. −0.16 (0.85), *p* < 0.001) during the acute disease. Higher values in the pulmonary pattern were also associated with abnormal pulmonary function testing (diffusing capacity of carbon monoxide or total lung capacity below 80% of expected and FEV1 to FVC ratio below 0.7). The cardiac, anosmia and headache patterns were associated with age younger than 65 years (0.06 (1.05) vs. −0.28 (0.63); 0.03 (1.04) vs. −0.17 (0.74); and 0.04 (1.04) vs. −0.19 (0.72), respectively, *p* < 0.001 for all comparisons). The pain-syndrome, pulmonary and cardiac patterns were inversely correlated with the time elapsed between the acute disease and clinic visit (Spearman’s rho −0.149, −0.122, and −0.225, respectively, *p* < 0.001 for all comparisons).

High values on the cognitive-emotional and cardiac patterns were associated with failure to return to baseline occupational status (mean 0.49 (SD 1.26) vs. −0.04 (0.99) *p* < 0.001; 0.44 (1.29) vs. 0.02 (0.99), *p* = <0.001 respectively). These two patterns were also associated with worsened physical activity status compared to pre-infection state (0.18 (1.10) vs. −0.09 (0.94), *p* < 0.001; 0.18 (1.18) vs. −0.08 (0.91), *p* = <0.001, respectively), while the anosmia pattern showed an inverse association with physical activity status compared to pre-infection state, implying improved physical status (−0.12 (1.02) vs. 0.06 (0.99), *p =* 0.007). These associations are presented in Table 3.

## 4. Discussion

In this cohort of individuals with long COVID, most of whom were in a non-severe acute phase of the disease, we identified six patterns of symptoms, including cognitive; pain syndrome; pulmonary; cardiac; anosmia-dysgeusia; and headache. Each pattern was associated with distinct symptoms of the acute illness; and had different implications on the return to work and to everyday functionality.

Mechanisms presumably involved in tissue damage in COVID-19 include direct viral invasion of the tissues, disorders during acute infection (e.g., thromboembolism, microvascular damage), and post-infectious immune responses. All of these are considered possible mechanisms for persistent organ damage leading to long COVID symptoms and may involve a specific system (e.g., the nervous system) [10], which might explain clustering of symptoms (e.g., the cognitive-emotional-fatigue cluster/pattern).

The pulmonary pattern in our study was linked to prior lung disease and the acute disease severity (hospitalization and WHO classifications of severe and critical COVID-19) and is probably the result of damage to the lung parenchyma itself and the endothelium. This pattern is similar to sequelae described following severe acute respiratory syndrome (SARS), Middle East respiratory syndrome (MERS) and other causes of viral pneumonitis [11,12]. Disease severity was also linked to the pain-syndrome pattern, and may reflect, at least in part, a post-ICU syndrome, combining paresthesia and fatigue, which may represent muscle weakness [13].

Neuro-psychiatric sequelae were also reported following SARS and MERS; however, these were mostly post-traumatic stress disorder (PTSD) and neurological dysfunction following intensive care treatment [14,15]. In our cohort, the cognitive-emotional pattern was not related to the severity of the acute phase of the disease and, in fact, was seen more often in persons who were not hospitalized. We can raise different hypotheses as to the causes of the cognitive-emotional pattern: micro-thrombi, direct invasion of the brain by the virus or elevated cytokines [16]. Cognitive impairments could be induced or exaggerated by emotional disturbances, as has been consistently observed in various psychiatric disorders [17]. This pattern’s association with failure to return to baseline employment status even at a median of four months following mild-moderate COVID-19, and its lack of association with time elapsed from the acute disease (hinting it does not decrease over time) suggests that this may become the true hallmark of long COVID burden on the individuum and the society.

Persistent fatigue, the main symptom described in all long COVID studies, was found to distribute equally in two main patterns (the cognitive and the pain syndrome patterns) and to trend highly in a third (the cardiac pattern). This finding is reminiscent of the two main examples of lasting fatigue: chronic fatigue syndrome and fibromyalgia [15,18], sometimes attributed to viral illnesses such as Epstein-Barr virus, SARS and Chikungunya, among others [19]. Fatigue is the only symptom exhibiting this type of distribution among the patterns. This distribution may imply that there are multiple etiologies of fatigue (physical, mental, emotional) and that, therefore, fatigue should be researched according to the accompanying symptoms or more specific features.

The cardiac (palpitations-chest pain) pattern is suggestive of pericardial or myocardial involvement and, interestingly, was inversely associated with age, and prior diagnosis of atrial fibrillation, suggesting an immune or a dysregulatory process, rather than an ischemic one. Peri-myocarditis has been described as part of acute COVID and in post-COVID patients [20]. Myocardial injury was found in outpatients recovering from mild disease on magnetic resonance imaging (MRI) of the heart, regardless of ongoing symptoms [21]. It is yet to be determined whether this is a result of direct viral involvement or immune-mediated damage.

Persistent anosmia and dysgeusia have been described and can be detrimental for patients’ quality of life. Our findings support the hypothesis that the mechanism for this phenomenon is viral infection of the nasal epithelium [22], rather than generalized illness or viral brain invasion, which may explain its appearance as an isolated symptom independent of the neuro-psychiatric and other patterns.

Long COVID symptoms in children have also been reported, albeit to a lesser extent than in adults [23,24]. A similar factor analysis may be beneficial in the pediatric population as well.

This study has several limitations. First, the sample might not be representative of all patients with complaints following the acute disease; however, the symptoms correlation matrix was probably not biased by that. Second, the data were collected from four active COVID centers, three during a clinic visit and the fourth, a telephone interview; questioning patients in four different countries and different languages, using different interview methods might create an information bias. In addition, data regarding pre-COVID status was lacking, hampering comparisons of post-COVID data (such as pulmonary function tests and physical activity status). Lastly, patients were recruited at different intervals post-acute COVID with a median of four months. This reflects real life data but might affect the factor analysis, as the six patterns might behave differently over time. This is reflected in the finding that the pain-syndrome, pulmonary and cardiac patterns seem to wane over time.

Our study suggests implications for clinical management. In the framework of a multi-disciplinary clinic, we recommend using a pattern-based approach, enabling management to focus on the most debilitating sets of symptoms in each patient. Patients suffering mainly from mental impairment would benefit from occupational rehabilitation and emotional support. Those with involvement of the lungs need dedicated follow-up and pulmonary rehabilitation. Patients with the cardiac pattern should be evaluated for risk of myocardial injury before returning to physical activity and those with anosmia-dysgeusia, fatigue and pain-syndrome patterns should be treated symptomatically and avoid unnecessary investigations. This is crucial given the colossal numbers of infected individuals and the limited capacity of health systems globally.

The study also has implications for research. The cognitive impairments are not explained by the severity of the acute disease; an effort should be made to identify the underlying mechanism, both through structural and functional imaging, and by investigating possible humoral explanations. Individuals with a predominant pulmonary pattern should be considered for clinical trials aimed at avoiding fibrotic disease and the cardiac pattern should be investigated in order to identify whether it is indeed rooted in cardiac injury and associated with long term impact on cardiovascular morbidity. Individuals suffering from the pain-syndrome pattern should be entered into long-term follow-up studies to identify optimal management strategies; patients with anosmia or dysgeusia should be evaluated for chemosensory research. This approach may enable an improved understanding of the pathophysiology and enable better allocation for future clinical trials.

## Figures and Tables

**Table 1:** Comparison of patient characteristics from the different centers.

	All	Israel	Switzerland	Spain	Italy
No. of patients, N (%)	1027	544 (53)	256 (24.9)	115 (11.2)	112 (10.9)
Gender–women, N (%)	559 (54.4)	307 (56.4)	144 (56.3)	60 (52.2)	48 (42.9)
Age, mean (SD)	49.2 (16.1)	46.4 (15.5)	45.5 (15.1)	58.4 (12.4)	61.2 (15.5)
BMI, mean (SD) Obese, N (%) * missing 280	28 (5.6) 223 (30.2) N = 738	27.6 (5.6) 149 (29.0) N = 518	28.7 (7.7) 3 (15.7) N = 19	29.8 (6.1) 42 (42.4) N = 99	28.2 (4.6) 29 (26.1) N = 111
Smoking, N (%) * missing 79	255 (24.8)	132 (24.3)	59 (23.0)	41 (35.7)	23 (20.5)
Diabetes, N (%)	92 (9.0)	45 (8.3)	10 (3.9)	22 (19.1)	15 (13.4)
Hypertension, N (%)	206 (20.1)	89 (16.4)	27 (10.5)	50 (43.5)	40 (35.7)
IHD, N (%)	52 (5.1)	25 (4.6)	17 (6.6)	1 (0.9)	9 (8.0)
Prior lung disease, N (%)	79 (7.7)	35 (6.4)	19 (7.4)	16 (13.9)	9 (8.0)
Hospitalization, N (%) * Missing 78	337 (32.8)	122 (22.4)	26 (10.2)	79 (68.7)	112 (100)
Severe disease ^a^, N (%)	264 (25.7)	86 (15.8)	26 (10.2)	72 (62.6)	80 (71.4)
Time to clinic visit (days), median (IQR) * Missing 26	123 (80–204)	97 (66–130)	217 (203–233)	115 (87–224)	146 (127–185)
Fatigue ^b^, N (%)	691 (67.2)	411 (75.6)	129 (50.4)	90 (78.3)	61 (54.5)
Dyspnea ^b^, N (%)	480 (46.7)	277 (50.9)	62 (24.2)	85 (73.9)	56 (50.0)
Cough ^b^, N (%)	187 (18.2)	108 (19.9)	21 (8.2)	35 (30.4)	23 (20.5)
Chest pain ^b^, N (%)	204 (19.9)	166 (30.5)	14 (5.5)	24 (20.9)	0 (0)
Palpitations ^b^, N (%)	104 (10.1)	67 (12.3)	18 (7.0)	19 (16.5)	0 (0)
Myalgia ^b^, N (%)	332 (32.3)	204 (37.5)	37 (14.5)	46 (40.0)	45 (40.2)
Arthralgia ^b^, N (%)	157 (15.3)	51 (9.4)	23 (9.0)	33 (28.7)	50 (44.6)
Parasthesias ^b^, N (%)	193 (18.8)	109 (20.0)	13 (5.1)	32 (27.8)	39 (34.8)
Insomnia ^b^, N (%)	259 (25.2)	204 (37.5)	27 (10.5)	28 (24.3)	0 (0)
Headache^b^, N (%)	153 (14.9)	69 (12.7)	44 (17.2)	24 (20.9)	16 (14.3)
Memory loss ^b^, N (%)	315 (30.7)	201 (36.9)	35 (13.7)	33 (28.7)	46 (41.1)
Concentration impairment ^b^, N (%)	312 (30.4)	212 (39.0)	31 (12.1)	26 (22.6)	43 (38.4)
Anosmia/dysguesia ^b^, N (%)	289 (28.2)	160 (29.4)	70 (27.3)	24 (20.9)	30 (26.8)
Worse physical activity status ^c^, N (%) * missing 42	326 (31.7)	142 (26.1)	134 (52.3)	43 (37.4)	7 (6.3)
Worse employment status ^c^, N (%) * missing 292	131 (12.8)	102 (18.8)	----	22 (19.1)	7 (6.3)
Pulmonary function testing performed, N (%)	665 (64.8)	517 (95.0)	----	51 (44.3)	97 (86.6)

Abbreviations: BMI—body mass index; IHD—ischemic heart disease, IQR—interquartile range, SD—standard deviation. ^a^: According to WHO severity grading—classified as severe and critical [8]. ^b^: Prevalence of persistent symptom at clinic visit, regardless of severity. ^c^: Compared to the pre-acute disease status. * Missing data on baseline characteristic.

**Table 2:** Patterns associations with selected demographics, comorbidities and features of the acute disease. Means for each of the patterns with standard deviation in parenthesis are shown. Significant associations ^a^ marked in bold. Analysis by *t*-test (Spearman where indicated).

	Feature N (%)	Cognitive-Emotional Pattern	Pain Syndrome Pattern	Pulmonary Pattern	Palpitations-Chest Pain Pattern	Anosmia-Dysgeusia Pattern	Headache Pattern
Gender	Women 559 (54.5)	0.08 (1.05)	**0.11 (1.08)**	0.03 (1.06)	0.04 (1.03)	0.05 (1.04)	**0.13 (1.13)**
	Men 466 (45.5)	−0.10 (0.93)	**−0.13 (0.88)**	−0.04 (0.93)	−0.05 (0.96)	−0.06 (0.94)	**−0.15 (0.79)**
Age	≥65 years 168 (16.4)	−0.17 (0.95)	0.09 (0.86)	0.06 (1.01)	**−0.28 (0.63)**	**−0.17 (0.74)**	**−0.19 (0.72)**
	<65 years 858 (83.6)	0.03 (1.01)	−0.02 (1.02)	−0.01 (1.00)	**0.06 (1.05)**	**0.03 (1.04)**	**0.04 (1.04)**
	Rho (*p*) ^b^	−0.05 (0.12)	**0.15 (<0.001)**	0.03 (0.34)	**−0.12 (<0.001)**	−0.06 (0.06)	−0.09 (0.006)
BMI	≥30 226 (30.2)	0.07 (1.09)	0.13 (0.95)	0.11 (1.09)	0.09 (1.07)	−0.06 (0.97)	−0.17 (0.98)
	<30 521 (69.8)	0.02 (1.02)	0.10 (0.99)	0.06 (0.99)	0.04 (1.02)	−0.02 (0.91)	−0.15 (0.86)
	Rho (*p*) ^b^	−0.02 (0.61)	0.04 (0.28)	0.04 (0.29)	0.01 (0.97)	−0.08 (0.03)	−0.05 (0.16)
Smoker (ever)	Yes 255 (26.9)	0.01 (1.04)	0.08 (1.04)	−0.02 (0.99)	0.04 (1.00)	0.00 (0.98)	−0.03 (0.98)
	No 693 (73.1)	0.02 (0.99)	−0.10 (0.93)	0.13 (1.05)	−0.05 (1.05)	0.00 (1.00)	−0.04 (1.04)
**Comorbidities**							
Prior lung disease	Yes 79 (7.7)	−0.01 (1.11)	−0.04 (0.94)	**0.41 (1.24)**	−0.08 (0.82)	−0.10 (1.02)	−0.08 (1.00)
	No 948 (92.3)	0.00 (0.99)	0.00 (1.01)	**−0.03 (0.97)**	0.01 (1.01)	0.01 (1.00)	0.01 (1.00)
Diabetes	Yes 92 (9.0)	−0.03 (1.03)	0.23 (0.97)	0.20 (1.14)	−0.01 (0.96)	−0.17 (0.80)	−0.27 (0.82)
	No 926 (91.0)	0.01 (1.00)	−0.02 (1.00)	−0.02 (0.99)	0.00 (1.01)	0.02 (1.02)	0.03 (1.02)
HTN	Yes 206 (20.2)	−0.07 (0.98)	0.10 (0.94)	0.12 (1.02)	−0.12 (0.82)	−0.10 (0.93)	**−0.21 (0.78)**
	No 812 (79.8)	0.02 (1.01)	−0.02 (1.02)	−0.03 (1.00)	0.04 (1.04)	0.03 (1.02)	**0.05 (1.05)**
IHD	Yes 52 (5.1)	0.14 (1.27)	−0.13 (0.77)	0.11 (1.05)	−0.22 (0.66)	−0.14 (1.02)	0.02 (1.25)
	No 966 (94.9)	−0.01 (0.99)	0.01 (1.01)	0.00 (1.00)	0.01 (1.02)	0.01 (1.00)	0.00 (0.99)
Atrial fibrillation	Yes 27 (2.6)	−0.33 (0.99)	0.12 (0.68)	0.00 (0.67)	**−0.33 (0.44)**	−0.33 (0.54)	−0.20 (0.72)
	No 1000 (97.4)	0.01 (1.00)	0.00 (1.01)	0.00 (1.01)	**0.01 (1.01)**	0.01 (1.01)	0.01 (1.01)
**Acute COVID-19 features**							
Hospitalization	Yes 337 (35.5)	**−0.15 (1.00)**	0.15 (0.95)	**0.17 (0.98)**	−0.11 (0.95)	**−0.16 (0.79)**	−0.17 (0.82)
	No 612 (64.5)	**0.10 (1.03)**	−0.02 (1.04)	**−0.07 (1.01)**	0.09 (1.08)	**0.07 (1.07)**	0.04 (1.07)
Abnormal CXR	Yes 140 (39.5)	−0.14 (1.05)	0.17 (0.96)	0.31 (1.06)	−0.04 (0.94)	−0.25 (0.72)	−0.10 (0.95)
	No 214 (60.5)	−0.07 (1.05)	0.40 (1.12)	0.12 (1.00)	0.04 (1.12)	0.04 (0.99)	−0.07 (0.92)
WHO severity	Severe 264 (25.6)	−0.16 (0.99)	**0.22 (0.98)**	**0.20 (1.07)**	−0.08 (0.94)	**−0.19 (0.78)**	−0.13 (0.86)
	Non-severe 763 (74.4)	0.05 (1.00)	**−0.08 (1.00)**	**−0.07 (0.97)**	0.03 (1.02)	**0.07 (1.06)**	0.05 (1.04)
**Symptoms of the acute disease**							
Fatigue ^c^	Yes 529 (69.0)	**0.25 (1.13)**	0.19 (1.08)	0.12 (1.09)	**0.22 (1.12)**	0.00 (1.02)	−0.17 (0.97)
	No 209 (31.0)	**−0.37 (0.75)**	0.03 (0.88)	0.04 (0.98)	**−0.19 (0.87)**	−0.03 (0.80)	−0.11 (0.78)
Dyspnea ^c^	Yes 405 (54.5)	0.12 (1.13)	0.24 (1.09)	**0.33 (1.16)**	0.19 (1.15)	−0.03 (0.98)	−0.14 (0.97)
	No 338 (45.5)	−0.02 (0.99)	0.04 (0.94)	**−0.16 (0.85)**	−0.01 (0.96)	0.01 (0.94)	−0.15 (0.86)
Anosmia ^c^	Yes 360 (49.2)	**0.34 (1.17)**	0.13 (1.05)	0.12 (1.09)	0.21 (1.04)	**0.36 (1.16)**	−0.23 (0.92)
	No 371 (50.8)	**−0.18 (0.90)**	0.15 (1.00)	0.09 (1.04)	0.00 (1.10)	**−0.34 (0.54)**	−0.05 (0.93)
Myalgia ^c^	Yes 418 (56.6)	**0.29 (1.18)**	**0.37 (1.15)**	0.16 (1.16)	0.19 (1.03)	0.02 (1.01)	−0.14 (1.06)
	No 321 (43.4)	**−0.22 (0.84)**	**−0.14 (0.75)**	0.03 (0.92)	−0.02 (1.10)	−0.05 (0.89)	−0.15 (0.71)
Time to clinic visit rho (*p*)	N = 1001	−0.04 (0.204)	**−0.149 (<0.001)**	**−0.122** **(<0.001)**	**−0.255** **(<0.001)**	0.032 (0.316)	**0.163** **(<0.001)**

Abbreviations: BMI—body mass index; CXR—chest radiogram; HTN—hypertension; IHD—ischemic heart disease; WHO—World Health Organization; ^a^: *p*-value < 0.001; ^b^: Spearman’s correlation with the continuous variable; ^c^: Symptoms of acute COVID-19 per description at clinic visit or telephone interview, regardless of severity.

**Table 3:** Patterns associations with patient outcomes at clinic visit. Means for each of the patterns with standard deviation in parenthesis are shown. Significant associations (*p* < 0.05) marked in bold. Analysis by *t*-test (Spearman where indicated).

	Feature N (%)	Cognitive-Emotional Pattern	Pain Syndrome Pattern	Pulmonary Pattern	Palpitations-Chest Pain Pattern	Anosmia-Dysgeusia Pattern	Headache Pattern
Failure to return to physical activity	Yes 326 (33.1)	**0.18 (1.10)**	0.07 (1.14)	0.08 (1.18)	**0.18 (1.18)**	**−0.12 (1.02)**	0.09 (1.18)
	No 659 (66.9)	**−0.09 (0.94)**	−0.02 (0.94)	−0.02 (0.97)	**−0.08 (0.91)**	**0.06 (0.99)**	−0.05 (0.90)
Failure to return to full employment	Yes 131 (17.8)	**0.49 (1.26)**	0.14 (1.12)	0.26 (1.27)	**0.44 (1.29)**	−0.02 (1.01)	−0.02 (1.13)
	No 604 (82.2)	**−0.04 (1.00)**	0.15 (1.00)	0.06 (1.00)	**0.02 (1.00)**	−0.02 (0.93)	−0.16 (0.89)
DLCO	Abnormal ^a^ 178 (26.7)	0.13 (1.15)	0.14 (0.90)	**0.38 (1.18)**	0.16 (1.21)	−0.09 (0.93)	−0.05 (1.01)
	Normal 487 (73.3)	0.03 (1.01)	0.07 (0.95)	**−0.04 (0.94)**	0.08 (1.00)	−0.02 (0.92)	−0.24 (0.82)
	Rho, (*p*) ^b^	−0.03 (0.43)	−0.09 (0.02)	**−0.18 (<0.001)**	−0.05 (0.23)	**0.13 (<0.001)**	0.01 (0.89)
TLC	Abnormal ^a^ 95 (14.5)	−0.07 (1.10)	0.25 (1.00)	**0.29 (1.11)**	0.01 (0.85)	−0.14 (0.87)	−0.19 (0.93)
	Normal 562 (85.5)	0.08 (1.05)	0.06 (0.92)	**0.02 (1.00)**	0.12 (1.10)	−0.02 (0.93)	−0.19 (0.87)
	Rho, (*p*) ^b^	0.3 (0.46)	−0.4 (0.29)	−0.09 (0.02)	−0.06 (0.16)	0.08 (0.06)	0.05 (0.25)
FeV1 to FVC ratio	Abnormal ^c^ 24 (3.6)	0.12 (1.05)	−0.01 (0.99)	**0.67 (1.30)**	0.23 (1.28)	0.07 (1.21)	0.14 (1.07)
	Normal 643 (96.4)	0.06 (1.05)	0.10 (0.93)	**0.04 (1.00)**	0.10 (1.06)	−0.04 (0.93)	−0.21 (0.87)
	Rho, (*p*) ^b^	−0.16 (<0.001)	0.11 (0.01)	0.01 (0.94)	−0.15 (<0.001)	0.11 (0.01)	0.11 (0.01)

Abbreviations: DLCO—Diffusing lung capacity of carbon monoxide; FeV1—Forced expiratory volume in 1 s; FVC—forced vital capacity; TLC—Total lung capacity. ^a^ Below 80% of expected; ^b^ Spearman’s correlation with the continuous variable; ^c^ A ratio of 0.7 and below.

## Data Availability

Data will be available upon reasonable request from the corresponding author.

## Supplementary Materials

The following supporting information can be downloaded at: https://www.mdpi.com/article/10.3390/jcm11040898/s1, Patient recruitment and data collection per country; Figure S1: Participant flow diagram; Table S1: Rotated Component Matrix—Table showing the loading of each symptom in each factor (pattern).
