# Supplementary material for: Patterns of Long COVID Symptoms: A Multi-Center Cross Sectional Study
Source: J Clin Med. 2022 Feb 9;11(4):898. doi: 10.3390/jcm11040898 (PMC8875229; doi:10.3390/jcm11040898)
Supplement: Supplementary file 1 [file jcm-11-00898-s001.zip › jcm-1574619-supplementary.pdf]

Supplemental Materials:

Patient recruitment and data collection per country:

Israel

Adult patients were consecutively recruited from a COVID-19 recovery clinic at Rabin Medical Center, Beilinson Hospital. During their visit, all participants were evaluated by an infectious diseases physician, a pulmonologist, and a social worker; and underwent pulmonary function testing and blood tests for SARS-CoV-2 serology. Prior to their visit, patients completed a follow-up chest radiogram, that was assessed at clinic visit by the pulmonologist.

During patient interview, patients were questioned regarding baseline characteristics, symptoms and duration of the acute disease, and ongoing symptoms according to a pre-planned questionnaire, grading the symptoms to 0 – none, 1- mild, 2 – moderate, 3 – severe.

Switzerland

Adult patients tested positive for SARS-CoV-2 infection (RT-PCR) at the Geneva University Hospitals, were contacted for elective remote ambulatory follow-up during the acute phase of the disease (first 10 days) and again at regular intervals. During the phone interview, participants were asked about baseline characteristics, symptoms and duration, as well as grading of symptoms when present from mild, moderate to severe.

Spain

Adult patients were recruited at Bellvitge University Hospital in Hospitalet de Llobregat, and at several Primary Care centers also at l'Hospitalet de Llobregat, El Prat de Llobregat and Gavà, which are all located in Barcelona, Spain

At Bellvitge Univesity Hospital, COVID-19 patients follow-up was performed to all patients who required admission to the hospital because of severe COVID-19 pneumonia and also to outpatients with COVID-19 disease who were initially assessed by Primary Care doctors and who presented new or worsened dyspnea or persistent infiltrates on chest X-ray, and were derived to the hospital. All these patients were initially evaluated telephonically by a pulmonologist with a chest X-ray. During the patients' interview, patients were questioned regarding baseline characteristics, symptoms, and ongoing symptoms according to a pre-planned questionnaire, grading the symptoms to 0 – none, 1- mild, 2 – moderate, 3 – severe. Based on the patients' symptomatology and the chest X-ray findings, it was considered to perform complementary studies (blood test, pulmonary function tests, 6-minute walking test, high-resolution computed tomography (HRCT) of the chest, transthoracic echocardiography and pulmonary gammagraphy), in addition to a joint face-to-face visit by pulmonologist and rehabilitation physician who ultimately decided the most appropriate treatment.

At the Primary Care centers, an initial advanced search was carried out in collaboration with the health technicians of the Primary Care service of the Llobregat Delta to identify the patients that were diagnosed with any of the inclusion criteria. Afterwards, the medical history of all the patients potentially eligible

for the study were carefully evaluated. Patients who met the inclusion criteria were contacted by phone, and the informed consent was requested.

All the included participants completed the pre-planned questionnaire, grading the symptoms to 0 – none, 1- mild, 2 – moderate, 3 – severe. In most cases, a face-to-face visit was required based on the evaluation of the questionnaire, and additional complementary explorations and assessment were performed as required.

Italy

Patients consecutively discharged from the University Hospital of Pisa with PCR-proven diagnosis of SARS-CoV-2 were actively recruited for follow-up. Baseline characteristics and data on acute phase were collected during the hospital stay; one month after discharge patients received a telephone questionnaire and three months after discharge they were invited to undergo: pulmonary visit, spirometry, plethysmography, DLCO, ABG analysis (if SpO2 < 95%), chest CT, chest ultrasound, blood test.

Table S1: Rotated Component Matrix—Table showing the loading of each symptom in each factor (pattern).

Symptom	Pattern					
	1	2	3	4	5	6
Headache	0.130	0.156	0.030	0.166	0.017	0.831
Anosmia	0.098	0.061	−0.005	0.034	0.949	0.004
Cough	0.100	0.082	0.840	−0.096	0.101	0.128
Dyspnea	0.078	0.199	0.659	0.270	−0.149	−0.132
Chest pain	0.241	0.003	0.432	0.466	0.024	0.037
Palpitations	−0.040	0.015	0.034	0.831	0.042	0.149
Paresthesia	0.210	0.575	0.209	0.058	0.132	−0.030
Memory impairment	0.775	0.246	0.070	−0.004	0.159	0.034
Concentration difficulty	0.766	0.204	0.118	−0.007	0.168	−0.018
Myalgia	0.299	0.704	0.076	0.158	0.034	−0.031

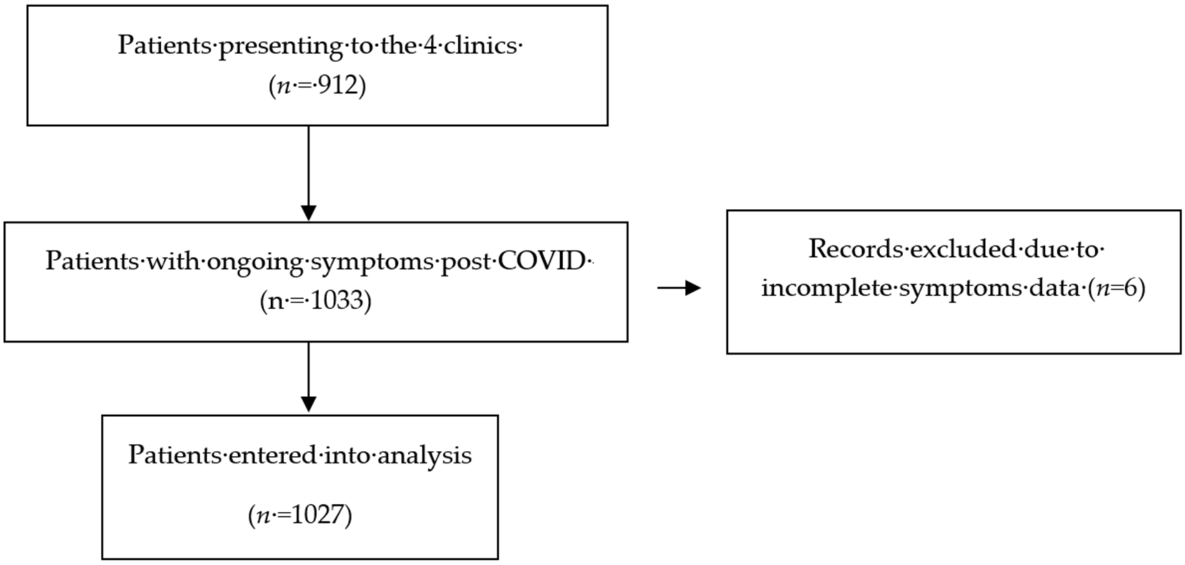

Figure S1. Participant flow diagram.
